# Transcriptomic insights into exercise-induced trabecular bone microarchitectural adaptations following combined aerobic and resistance training in mice

**DOI:** 10.1038/s41598-026-56753-6

**Published:** 2026-06-15

**Authors:** Jiyeon Kim, Seungyong Lee, Jinkyung Cho, Hyo Youl Moon, Hee-Jung Park, Dong-Ho Park, Changsun Kim

**Affiliations:** 1 Department of Cardiovascular & Metabolic Sciences, Cleveland Clinic Research, Cleveland, OH USA; 2 Department of Physical Education, Incheon National University, Incheon, Korea; 3 College of Sport Science, Sungkyunkwan University, Suwon, Korea; 4 Department of Physical Education, Seoul National University, Seoul, Korea; 5 Department of Biomedical Science and Engineering, Inha University, Incheon, Korea; 6 Department of Kinesiology, Inha University, Incheon, Korea; 7 Department of Physical Education, Dongduk Women’s University, 60 Hwarang-ro13-gil, Sungbuk-gu, 02748 Seoul, South Korea

**Keywords:** Cell biology, Diseases, Genetics, Medical research

## Abstract

Osteoporosis is a prevalent musculoskeletal disorder, rising in incidence and impact as the global population ages. Peak bone mass (PBM), determined by bone mineral density (BMD) during adolescence, is a key determinant of skeletal health and later osteoporosis risk. Exercise enhances BMD, yet its molecular mechanisms remain unclear. This study examined combined exercise effects on bone health in early adult mice using RNA sequencing (RNA-seq) analysis. Nineteen-week-old mice were randomly assigned to control (CON, n=8) or combined exercise (EXE, n=8) groups. The 12-week intervention included aerobic and resistance training, with physical performance tests conducted before and after. Following intervention, tibial bone characteristics were assessed by dual-energy X-ray absorptiometry (DXA) and micro-computed tomography (μCT), while femoral gene expression was analyzed using transcriptomic analysis. EXE mice demonstrated significant increases in grip strength and exhaustion test performance, but not in the rotarod test. Proximal tibial trabecular bone microarchitecture was enhanced in the EXE group, with increased bone volume fraction (BV/TV), trabecular thickness (Tb.Th), and trabecular number (Tb.N), along with a trend toward reduced trabecular separation(Tb.Sp). Transcriptomic analysis revealed 109 upregulated and 551 downregulated differentially expressed genes. Gene ontology analysis highlighted enrichment of terms related to muscle cell differentiation, contraction, and ion regulation. Bone metabolism-related GO Biological Process terms were specifically enriched, with Pax1 and Dcstamp upregulated and Fgf18, Scx, and Scube2 downregulated. KEGG analysis identified eleven significantly enriched pathways, including Calcium signaling, ECM-receptor interaction, and PI3K-Akt signaling. These findings suggest that combined exercise enhances trabecular bone microarchitecture and induces transcriptomic changes involving genes associated with bone development, remodeling, and extracellular matrix organization, providing molecular-level evidence for exercise-induced skeletal adaptation.

**Supplementary Information:**

The online version contains supplementary material available at https://doi.org/10.1038/s41598-026-56753-6.

## Introduction

Osteoporosis is a major musculoskeletal disorder characterized by low bone mass and the deterioration of bone microarchitecture, leading to an increased risk of fragility fractures^1, 2^. With advancing age, bone formation declines while bone resorption increases, primarily due to reduced osteoblast function and hormonal changes^3, 4^. As the global population continues to age, the prevalence and socioeconomic burden of osteoporosis are steadily rising^5^.

Meanwhile, adolescence and early adulthood represent critical periods for peak bone mass acquisition, a major determinant of long-term skeletal integrity in later life^6, 7^. During adolescence, bone tissue undergoes rapid growth and maturation, driven by increased osteoblastic activity, leading to the attainment of peak bone mass by early adulthood^8, 9^. Studies have shown that individuals who achieve sufficient bone mass by early adulthood are less susceptible to age-related bone loss and have a significantly reduced risk of developing osteoporosis and fractures later in life^10^. Therefore, adolescence and early adulthood are considered a critical “window of opportunity” for the primary prevention of osteoporosis, as bone mass accrued during this period serves as a biological reserve to mitigate future bone loss^11^.

Exercise is widely recognized as an effective non-pharmacological strategy to prevent osteoporosis by promoting bone remodeling and enhancing bone mechanical strength^12, 13^. Mechanical loading during physical activity stimulates osteoblast-driven bone formation and inhibits osteoclastic resorption, thereby improving bone mineral density (BMD) and bone quality^14^. In particular, aerobic exercise enhances systemic circulation and metabolic activity, thereby facilitating the delivery of nutrients and oxygen to bone tissue, which helps maintain bone remodeling balance^15^. Regular and appropriately intense aerobic exercise has been reported to moderately increase BMD and improve bone microarchitecture^16^. In contrast, resistance exercise provides a more direct mechanical stimulus to bone, robustly activating osteoblasts and promoting bone formation^17^. Resistance training is particularly effective in increasing BMD and inducing structural adaptations in both trabecular and cortical bone, thereby enhancing bone strength^18^.

Based on these findings, maximizing peak bone mass through a combination of aerobic and resistance exercise during early adulthood represents a crucial strategy for promoting bone health and preventing osteoporosis. While previous studies have examined transcriptomic responses to aerobic exercise alone in bone tissue of both healthy and disease models^19–21^, no study has investigated the bone transcriptome following combined aerobic and resistance exercise during the peak bone mass acquisition period in early adulthood. Therefore, this study aimed to investigate the effects of combined exercise on bone microarchitecture and gene expression in early adult mice using RNA sequencing analysis.

## Methods

### Animals

A total of 16 female C57BL/6J mice (8 weeks old) were purchased from Central Lab (Seoul, Korea) and reared until 17 weeks of age. Female mice were selected given that osteoporosis disproportionately affects women^1^. Additionally, 17 weeks of age was chosen to represent early adulthood in mice, a critical period for peak bone mass acquisition, as BMD in female C57BL/6J mice has been shown to peak at approximately 17 weeks of age^22^. The mice were housed at a maximum of five per standard cage with ad libitum access to rodent chow and tap water. All animals were acclimated to a 12-hour light/dark cycle, a controlled temperature (22 ± 2 °C), and humidity (50%) throughout the entire experimental period. Mice were anesthetized via intraperitoneal injection at the time of euthanasia with a freshly prepared mixture of Zoletil (tiletamine-zolazepam) and Rompun (xylazine) at a 3:1 ratio. The doses used were 30 mg/kg for Zoletil and 10 mg/kg for Rompun. The injection volume was approximately 40 µL per 10 g body weight. All experimental procedures and reporting in this study were conducted in compliance with the ARRIVE guidelines.

### Study design and exercise protocol

All mice were randomly divided into two groups: 1) a control group (CON, n=8), and 2) a combined exercise group (EXE, n=8). Body mass was measured weekly throughout the intervention period. The pre-intervention body mass was measured at 17 weeks of age, prior to the acclimation period, and the post-intervention body mass used for pre-versus-post comparison was measured at 29 weeks of age, corresponding to the end of week 11 of the formal 12-week training intervention. Physical performance tests were conducted pre- and post-exercise training intervention.

**Fig. 1:**
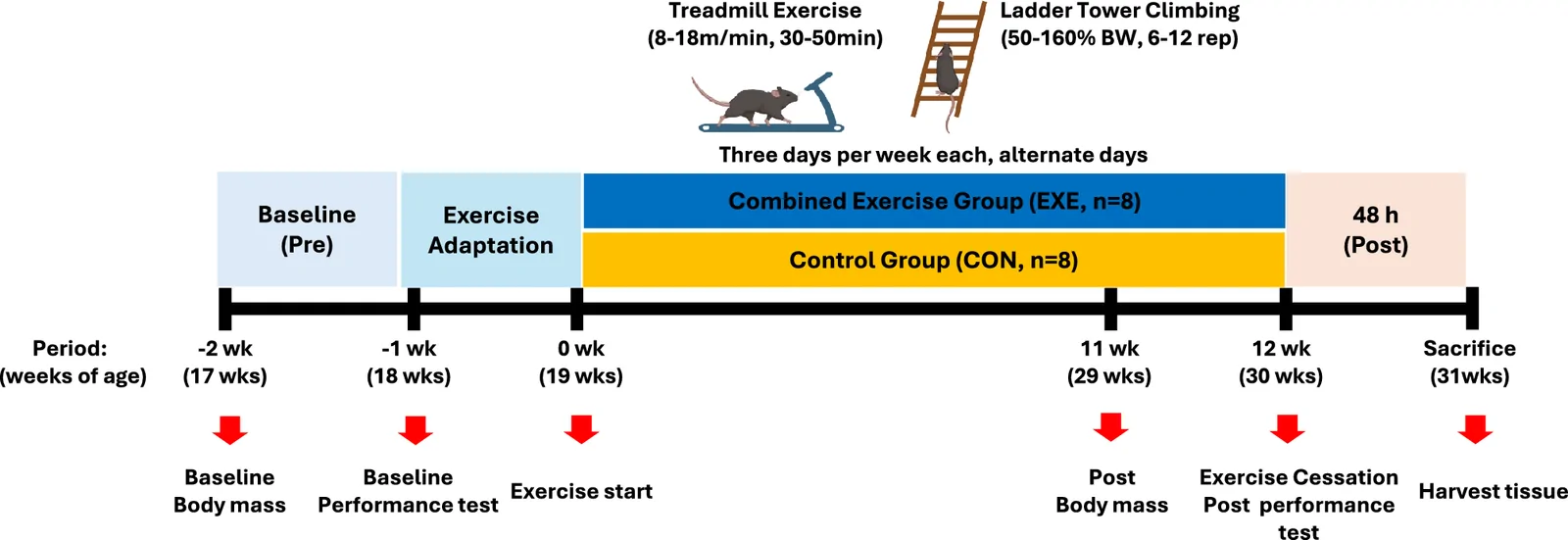
Experimental timeline and exercise training protocol.

The EXE group underwent a one-week acclimation period (18 weeks of age), after which the mice were gradually trained with increasing intensity and duration. The combined exercise training program consisted of both aerobic and resistance training. From 19 weeks of age, the EXE group performed treadmill running on a motorized rodent treadmill (JD-A-09, Jung Do B&P, Seoul, Korea) and ladder climbing exercises on a custom-built climbing ladder, three days per week each on alternate days, for a total of 12 weeks. Aerobic exercise training was performed on a motorized treadmill at 0° incline, with speeds ranging from 8 to 18 m/min for 20 to 50 minutes per session. Resistance training consisted of ladder climbing (height 90 cm, rung spacing 2 cm, angle 85°) with loads ranging from 50% to 160% of body weight, with 6 to 12 repetitions per session. Mice in the control group were home-cage controls and were not placed on the treadmill or ladder during the intervention period. The 30-week (week 12) body mass measurement was excluded from the pre-versus-post comparison because it was obtained during the post-intervention physical performance testing period, including an all-out exhaustion test, which induced acute, transient body mass changes unrelated to the training intervention itself. At 31 weeks of age, all mice were sacrificed 48 hours after the final exercise training session. An overview of the experimental exercise design is presented in (Fig. 1). Detailed aerobic treadmill exercise and ladder climbing resistance exercise protocols are presented in (Supplementary Tables 1 and 2).

The right tibiae were harvested at sacrifice and fixed in 4% paraformaldehyde at 4°C for 24 hours. Tibiae were subsequently stored in 70% ethanol at -20°C until getting scanned by dual-energy X-ray absorptiometry (DXA) and micro-computed tomography (μCT). The left femora were collected at sacrifice and stored in RNA stabilization solution (Invitrogen, AM7020) at -80°C for gene expression analysis. In addition, heart, visceral fat, liver, and skeletal muscles including gastrocnemius, soleus, and plantaris were harvested and weighed at the time of sacrifice to assess the physiological effects of the exercise intervention.

### Physical performance

- 1) Grip strength
  Grip strength was measured using a grip strength meter (BIO-GS3, Bioseb). Each mouse was allowed to grasp a metal grid with its forelimbs and was gently pulled horizontally by the tail with gradually increasing force until the applied force exceeded the animal’s grip strength. The peak force (in grams) generated during the pull was recorded. A total of six measurements were taken per animal, and the representative value was calculated as the mean after excluding the highest and lowest values.
- 2) Rota-rod test
  Motor coordination and balance were assessed using a Rota-rod apparatus (RRMR, BS Technology, Korea). Prior to the formal test, all mice, including both EXE and CON groups, underwent two habituation sessions on separate days to minimize the influence of novelty-induced stress on performance. During the formal test, the apparatus was set to an acceleration phase from 4 to 40 rpm over 1 minute, followed by a constant speed of 40 rpm. Each mouse underwent three trials with a maximum duration of 5 minutes per trial and a 1-minute rest period between trials. The average of the two longest durations was recorded as the representative value.
- 3) Exhaustion test
  The exhaustion test protocol was adapted from Ferreira et al.^23^ with modification. Prior to the formal test, CON group mice underwent treadmill familiarization on two separate days, as they had not been previously exposed to treadmill running during the intervention period. The treadmill was initiated at 6 m/min and speed was incrementally increased by 3 m/min every 3 minutes, up to a maximum of 33 m/min. Mice were subjected to continuous treadmill running until exhaustion, defined as the inability to remain off the shock grid for more than 5 consecutive seconds despite mechanical stimulation. Total running time (minutes) was recorded as the endpoint measure.

### DXA scan

Dual-energy X-ray absorptiometry (DXA) was performed using the UltraFocus DXA system (Faxitron Bioptics, LLC, Tucson, AZ, USA) equipped with small animal-specific analysis software, following tissue harvest. The entire tibia was scanned to evaluate bone area, bone mineral density (BMD), and bone mineral content (BMC). DXA data from one mouse per group (one CON and one EXE) were excluded because the tibiae sustained partial fractures during tissue processing, precluding accurate whole-bone scan analysis. The micro-computed tomography (μCT) analysis was still feasible for these samples, as the regions of interest (proximal metaphysis and mid-diaphysis) remained intact.

### μCT imaging and analyses

The tibia was dissected free of skin and muscle and analyzed using a high-resolution micro-computed tomography (μCT) system (SkyScan 1172, Bruker, Kontich, Belgium). Scans were obtained at an image resolution of 10 μm, with the following settings: 1 mm aluminum filter, 65 kVp X-ray voltage, 153 μA anode current, 65 ms exposure time, 5-frames averaging, and 0.3 degrees rotation step. The 2D X-ray projections were reconstructed into 3D images using the Feldkamp algorithm via NRecon software (v. 2.0.4.0, SkyScan). Subsequent 3D morphometric analyses were performed using CTVox and CTAn software (v. 1.13, SkyScan). Trabecular bone microarchitecture in the proximal tibial metaphysis and cortical bone parameters in the mid-diaphysis were assessed as previously described^19, 24^. Briefly, for trabecular bone analysis, 50 slices were made in the proximal tibial metaphyses, beginning at 500 μm inferior to the growth plate. Trabecular bone microarchitectural parameters including bone volume to tissue volume ratio (BV/TV, %), trabecular thickness (Tb.Th, μm), trabecular number (Tb.N, /mm^2^), and trabecular separation (Tb.Sp, μm) were calculated. For cortical bone parameters analyses, 50 slices at the tibial midshaft were selected to determine cortical bone volume (Ct.BV, mm^3^), cortical area (Ct.Ar, mm^2^), cortical thickness (mm), and polar moment of inertia (pMOI, mm^4^). To segment bone tissue from marrow and background, a global thresholding procedure was applied to the reconstructed grayscale images. The binarization threshold was set at a range of 50 to 255 for both trabecular bone and cortical bone.

### RNA sequencing and bioinformatic analysis

Total RNA was extracted from the left femora of both CON and EXE mice and submitted to Macrogen (Seoul, Korea) for transcriptomic analysis. RNA sequencing was performed using the SureSelectXT RNA Direct Library Preparation Kit on an Illumina platform with paired-end reads (101 bp). A total of 8 samples were sequenced (CON: n=4; EXE: n=4). Raw sequencing reads were quality-assessed using FastQC (v0.11.7). Low-quality bases and adapter sequences were trimmed using Trimmomatic (v0.38). Trimmed reads were aligned to the mouse reference genome (mm10, NCBI annotation 108) using HISAT2 (v2.1.0). Transcript assembly and expression quantification were performed using StringTie (v2.1.3b). Prior to differential expression analysis, genes with zero counts in any sample were excluded, retaining 20,126 genes. Data normalization was performed using Relative Log Expression (RLE) normalization via DESeq2. Sample quality was assessed using Multidimensional Scaling (MDS) analysis, which confirmed acceptable clustering of biological replicates within each group. All 8 samples (CON: n=4; EXE: n=4) were used for differential expression analysis. Differentially expressed genes (DEGs) were defined as those exhibiting a ≥2-fold change with p < 0.05. Functional enrichment analyses for Gene Ontology (GO) terms and KEGG pathways^25–27^ were performed using the g:Profiler tool (https://biit.cs.ut.ee/gprofiler/) with Mus musculus selected as the organism. Multiple testing correction was applied using the g:SCS algorithm with a significance threshold of adjusted p < 0.05. Hierarchical clustering was used to group samples based on DEG similarity, and results were visualized using heatmaps.

## Statistics

**Fig. 2:**
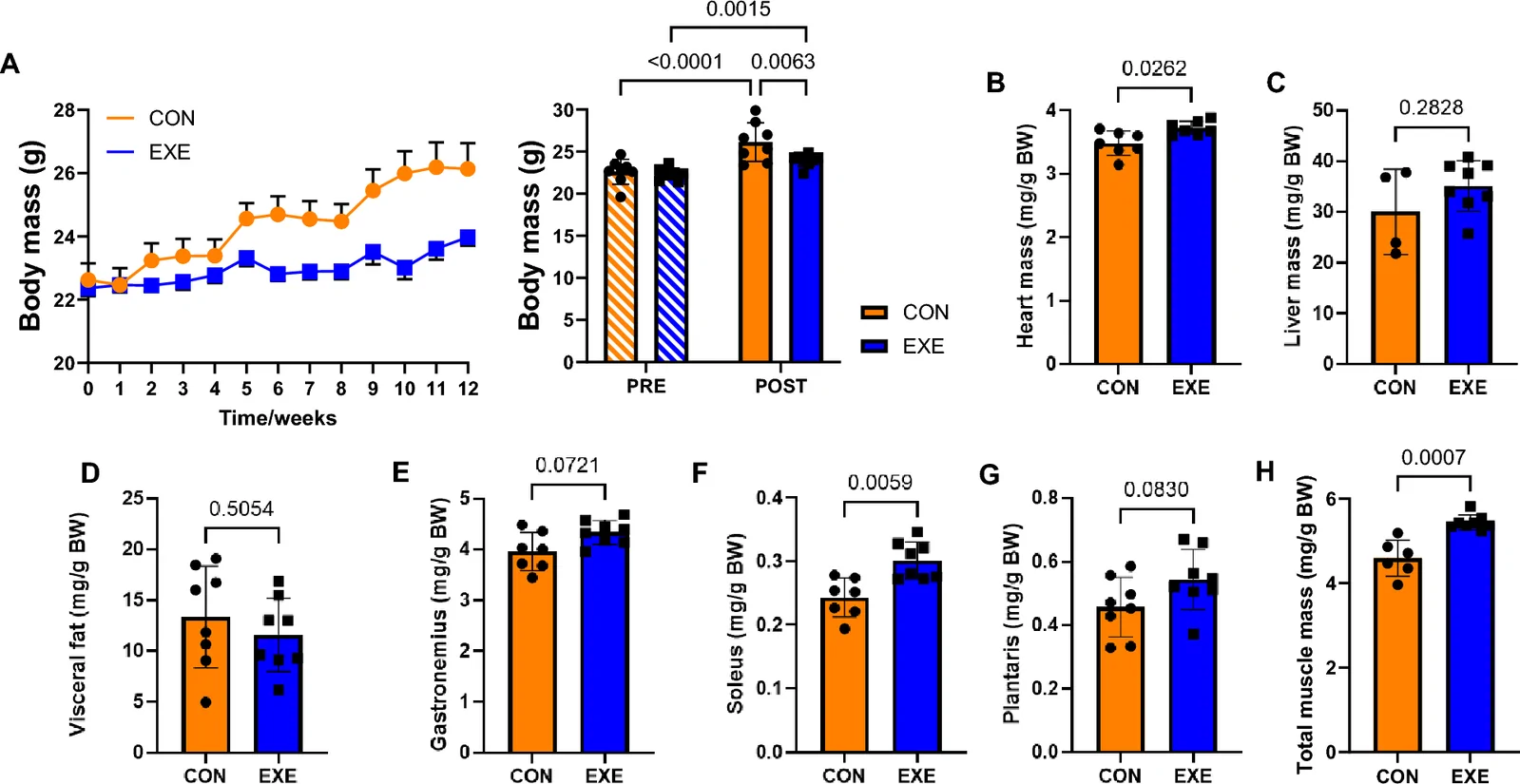
Body mass changes and tissue weights following 12-week combined exercise training. (**A**) Body mass over the 12-week intervention period (left) and comparison of pre- (17 weeks of age) versus post-intervention (29 weeks of age) body mass (right). Normalized tissue weights (mg/g body weight) are shown for (**B**) heart, (**C**) liver, (**D**) visceral fat, (**E**) gastrocnemius, (**F**) soleus, (**G**) plantaris, and (**H**) total skeletal muscle (sum of gastrocnemius, soleus, and plantaris). Body mass data in (**A**) were analyzed using two-way repeated-measures ANOVA followed by Šídák’s multiple comparisons test (line graph) or Fisher’s LSD test (bar graph). Tissue weight data (**B-H**) were analyzed using the Mann-Whitney test with outliers identified by the ROUT method (Q = 1%). Sample sizes: Heart (n=7/7), Liver (n=4/8), Visceral fat (n=8/8), Gastrocnemius (n=7/8), Soleus (n=7/8), Plantaris (n=8/8), Total muscle (n=6/8) for CON/EXE. Values are mean ± SE for the line graph in (**A**) and mean ± SD for the bar graph in (**A**) and panels (**B-H**).

Mice were initially assigned to two groups (n=8 per group), and this sample size was determined based on prior rodent exercise studies examining bone microarchitecture following treadmill-based exercise interventions^19, 28^, which demonstrated sufficient statistical sensitivity to detect exercise-induced changes in trabecular bone parameters with comparable or smaller group sizes (n=6-7 per group). For the RNA sequencing analysis, a subset of n=4 mice per group was randomly selected, consistent with sample sizes used in published rodent bone transcriptomic studies^20^. Tissue weights were normalized to body weight (mg/g body weight) prior to statistical comparison to account for exercise-induced differences in body mass. The final sample size for each normalized tissue weight comparison varied (n=4-8 per group) due to procedural omissions during tissue collection in a subset of CON mice and the exclusion of outliers identified by the ROUT method (Q = 1%) in GraphPad Prism (version 10, GraphPad Software, San Diego, CA, USA). Specific sample sizes for each tissue are reported in the Fig. 2 legend. Differences in normalized tissue weights and bone microarchitecture measurements between the CON and EXE groups were assessed using the Mann-Whitney test. Body mass over the 12-week intervention period was analyzed using two-way repeated-measures ANOVA followed by Šídák’s multiple comparisons test. Pre- versus post-intervention body mass comparisons and physical performance data (grip strength, exhaustion test, rota-rod) were analyzed using two-way repeated-measures ANOVA followed by Fisher’s LSD test for pre-planned comparisons. Quantitative data are expressed as mean ± SD, with p < 0.05 considered significant. For RNA sequencing data, differential expression analysis was performed using DESeq2 (R package) with the negative binomial Wald test (nbinomWaldTest). Genes with |fold change| ≥ 2 and raw p-value < 0.05 were considered differentially expressed. Hierarchical clustering was performed using Euclidean distance with complete linkage.

## Results

### Animal body mass and tissue weights following 12 weeks of combined exercise training

Body mass was measured weekly throughout the 12-week intervention. There were no significant differences between the control (CON) and exercise (EXE) groups at baseline (17 weeks of age, p=0.7229). Two-way repeated-measures ANOVA revealed a significant time × group interaction (p=0.0055), indicating that body mass trajectories differed between groups. Both groups gained body mass over the intervention period, but the magnitude of body mass gain was substantially attenuated in the EXE group (PRE: 22.36 g, POST: 23.98 g, increase of 1.61 g; p=0.0015) compared to the CON group (PRE: 22.63 g, POST: 26.14 g, increase of 3.51 g; p<0.0001). Consequently, at the post-intervention time point (29 weeks of age), body mass was significantly lower in the EXE group compared to the CON group (p=0.0063) (Fig. 2A). At sacrifice, organ weights (heart and liver), skeletal muscle weights (gastrocnemius, soleus, and plantaris), and visceral fat mass were measured and normalized to body weight (mg/g body weight) to account for exercise-induced differences in body mass.

Normalized heart mass was significantly higher in the EXE group compared to the CON group (p=0.0262) (Fig. 2B), while no significant differences were observed in normalized liver mass (p=0.2828) or visceral fat mass (p=0.5054) between groups (Fig. 2C, D). For skeletal muscles, normalized gastrocnemius mass showed a non-significant trend toward higher values in the EXE group (p=0.0721) (Fig. 2E), whereas normalized soleus mass was significantly higher in the EXE group (p=0.0059) (Fig. 2F), and normalized plantaris mass also showed only a non-significant trend (p=0.0830) (Fig. 2G). Notably, normalized total skeletal muscle mass (sum of gastrocnemius, soleus, and plantaris) was significantly greater in the EXE group compared to the CON group (p=0.0007) (Fig. 2H), indicating that combined aerobic and resistance training preserved or enhanced lean mass relative to body weight despite the attenuated body mass gain. The smaller increase in absolute body mass in the EXE group is therefore likely attributable to a reduction in body fat accumulation rather than a loss of lean tissue.

### Combined exercise training improves physical fitness in mice

**Fig. 3:**
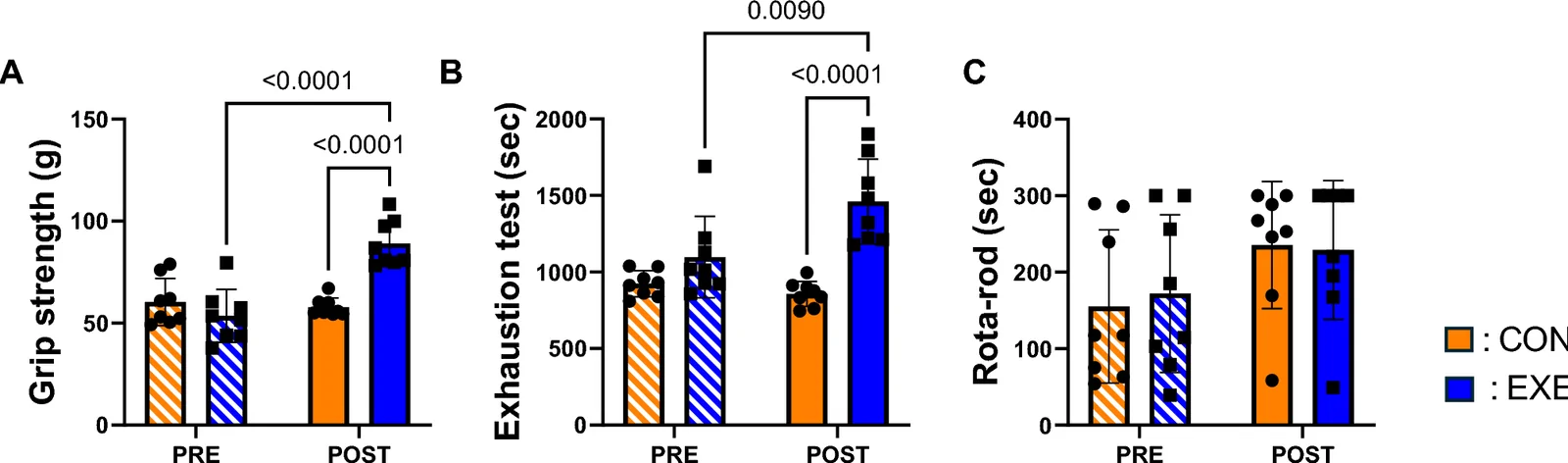
Physical performance measurements before and after 12-week combined exercise training. (**A**) Grip strength, (**B**) exhaustion test, and (**C**) rota-rod performance measured at pre- and post-intervention in the control (CON) and combined exercise (EXE) groups. Data were analyzed using two-way repeated-measures ANOVA followed by Fisher’s LSD test for pre-planned comparisons. n=8 per group. Values are mean ± SD.

Grip strength, an indicator of muscular strength, showed a significant time × group interaction (p<0.0001), indicating differential responses between groups over the intervention period. Grip strength significantly increased in the EXE group from pre- to post-intervention (53.57 to 89.01, a 66% increase; p<0.0001), whereas no significant change was observed in the CON group (60.39 to 57.85, p=0.5338). Consequently, post-intervention grip strength was significantly greater in the EXE group compared to the CON group (p<0.0001) (Fig. 3A).

Endurance capacity, assessed via the exhaustion test, also showed a significant time × group interaction (p=0.0241). The exhaustion time significantly increased in the EXE group from pre- to post-intervention (1098 to 1462 seconds, a 33% increase; p=0.0090), whereas no significant change was observed in the CON group (923 to 858 seconds, p=0.5938). Post-intervention exhaustion time was significantly greater in the EXE group compared to the CON group (p<0.0001) (Fig. 3B).

In contrast, performance in the rota-rod test, which evaluates motor coordination and balance, showed a significant time main effect (p=0.0161) with no significant time × group interaction (p=0.6505) or group main effect (p=0.8984). Both groups showed improvement over time, although the increase reached statistical significance only in the CON group (p=0.0401) and not in the EXE group (p=0.1302), with no significant differences between groups at either time point (Fig. 3C).

### Combined exercise training improves trabecular bone microarchitecture in mice

**Fig. 4:**
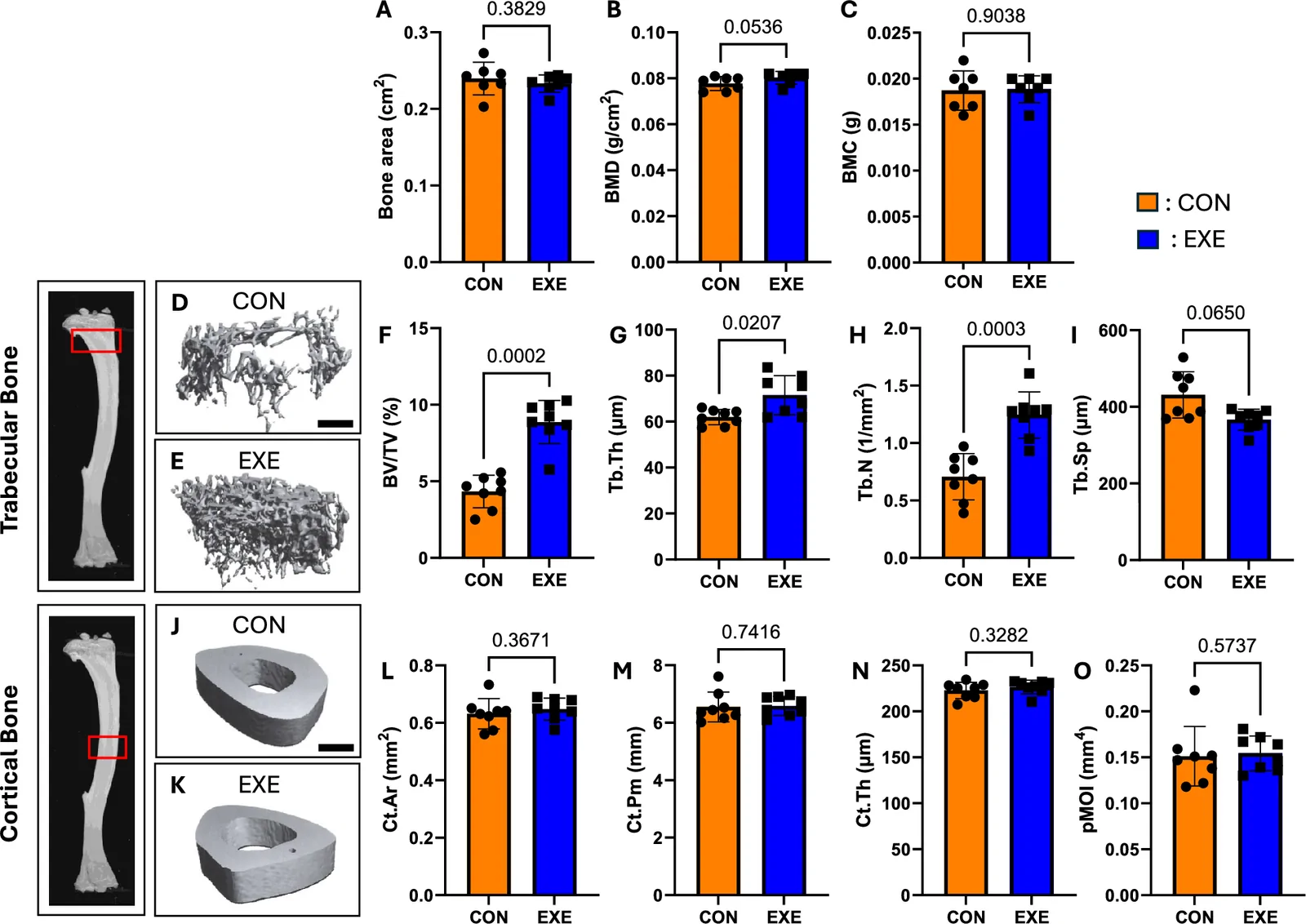
Proximal tibial bone mineral density, bone mineral content, trabecular bone microarchitecture, and cortical bone parameters following 12-week combined exercise training. Quantification of proximal tibial area, BMD, and BMC is shown in panels A–C. No significant differences were observed in (**A**) tibial area, (**B**) BMD, and (**C**) BMC between the exercise (EXE) and control (CON) groups. In contrast, trabecular bone microarchitecture differed significantly between the two groups as shown in the representative images (**D**, **E**). The EXE group exhibited higher (**F**) trabecular bone volume fraction (BV/TV), (**G**) trabecular thickness (Tb.Th) and (**H**) trabecular number (Tb.N), along with lower (**I**) trabecular separation (Tb.Sp), compared to the CON group. However, no significant differences were found in cortical bone parameters between groups as shown in J and K. (**L**) Cortical bone area (Ct.Ar), (**M**) cortical perimeter (Ct.Pm), (**N**) cortical thickness (Ct.Th), and (**O**) polar moment of inertia (pMOI) were not significantly different between the EXE and CON groups. Black scale bar: 1 mm. Statistical analysis was performed using the Mann-Whitney (non-parametric) test. For DXA analysis (panels **A-C**), n=7 per group due to exclusion of one mouse per group (one CON and one EXE) with partially fractured tibiae that precluded accurate whole-bone scan. For μCT analysis (panels F-I, L-O), n=8 per group. Data are presented as means ± SD.

We investigated whether a 12-week combined exercise training intervention increased bone mineral density (BMD), bone mineral content (BMC), trabecular bone microarchitecture, or cortical bone characteristics in the proximal tibia. Although no differences were observed in tibial area, BMD, and BMC values (Fig. 4), significant improvements in proximal tibial trabecular bone microarchitecture were observed in the EXE group compared to the CON group. Specifically, the trabecular bone parameters BV/TV, Tb.Th, and Tb.N were higher in the EXE group than in the CON group by 105%, 15.5%, and 76.4%, respectively (Fig. 4F, G, H). Conversely, Tb.Sp was 15% lower in the EXE group than in the CON group (Fig. 4I). Additionally, no significant differences were found in cortical bone parameters, including cortical area (Ct.Ar), cortical perimeter (Ct.Pm), cortical thickness (Ct.Th), and polar moment of inertia (pMOI) (Fig. 4L, M, N, O). These findings are consistent with previous studies demonstrating that exercise training improves trabecular bone microarchitecture, including increases in BV/TV, Tb.Th, and Tb.N, in rodent models^28^.

### Gene expression profiling and bioinformatics analysis

**Fig. 5:**
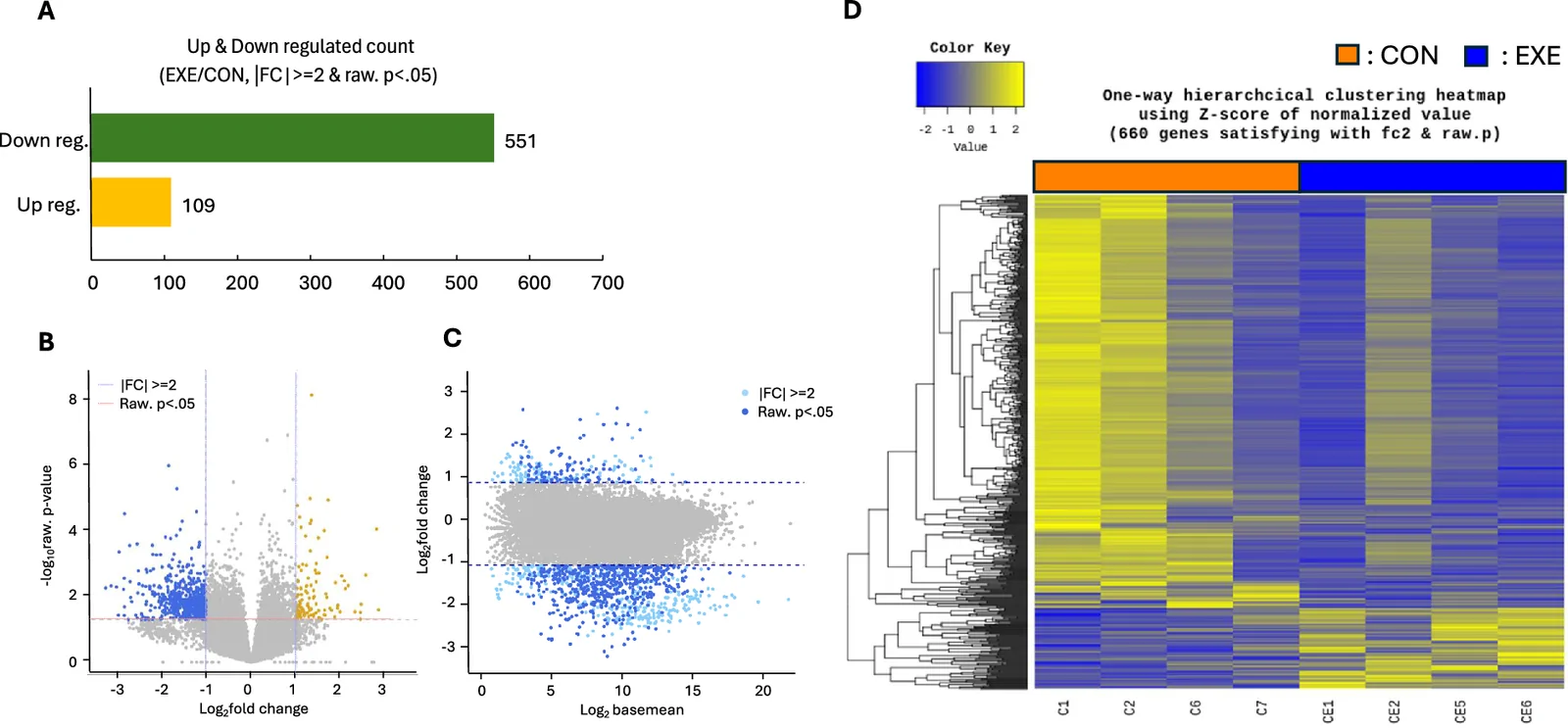
Overview of differentially expressed genes (DEGs) in bone tissue following 12-week combined exercise training. RNA sequencing-based transcriptomic analysis identified 660 DEGs (*p*<0.05) in the bones of early adult mice following combined exercise training, including 109 upregulated and 551 downregulated genes compared to controls (**A**). (**B**) The volcano plot and (**C**) MA plot highlight the most significantly altered gene expression profiles between the control and exercise groups. (**D**) A heatmap illustrates distinct clustering patterns of gene expression between the two groups.

To understand the effect of 12 weeks of combined exercise training on the bone tissue of early adult mice, we performed gene expression profiling and bioinformatics analysis. RNA sequencing revealed a total of 660 differentially expressed genes (DEGs) that exhibited significant changes (|fold change| ≥ 2 and raw *p*-value < 0.05) following exercise training (Fig. 5A). Among these, 109 genes were upregulated and 551 genes were downregulated compared to the control group. The volcano and MA plots clearly highlighted several genes with more than a two-fold difference in expression between groups (Fig. 5B, C). Hierarchical clustering of these DEGs revealed distinct expression patterns between the control and exercise groups, while gene expression profiles were highly consistent within each group (Fig. 5D).

Based on fold change values among the DEGs, keratocan was the most significantly down regulated gene following exercise training, followed by Ccl21a (chemokine (C-C motif) ligand 21A). Conversely, the most upregulated genes were members of the immunoglobulin kappa variable (Igkv) family and formyl peptide receptor 3 (Fpr3) (Supplementary Table 3).

**Fig. 6:**
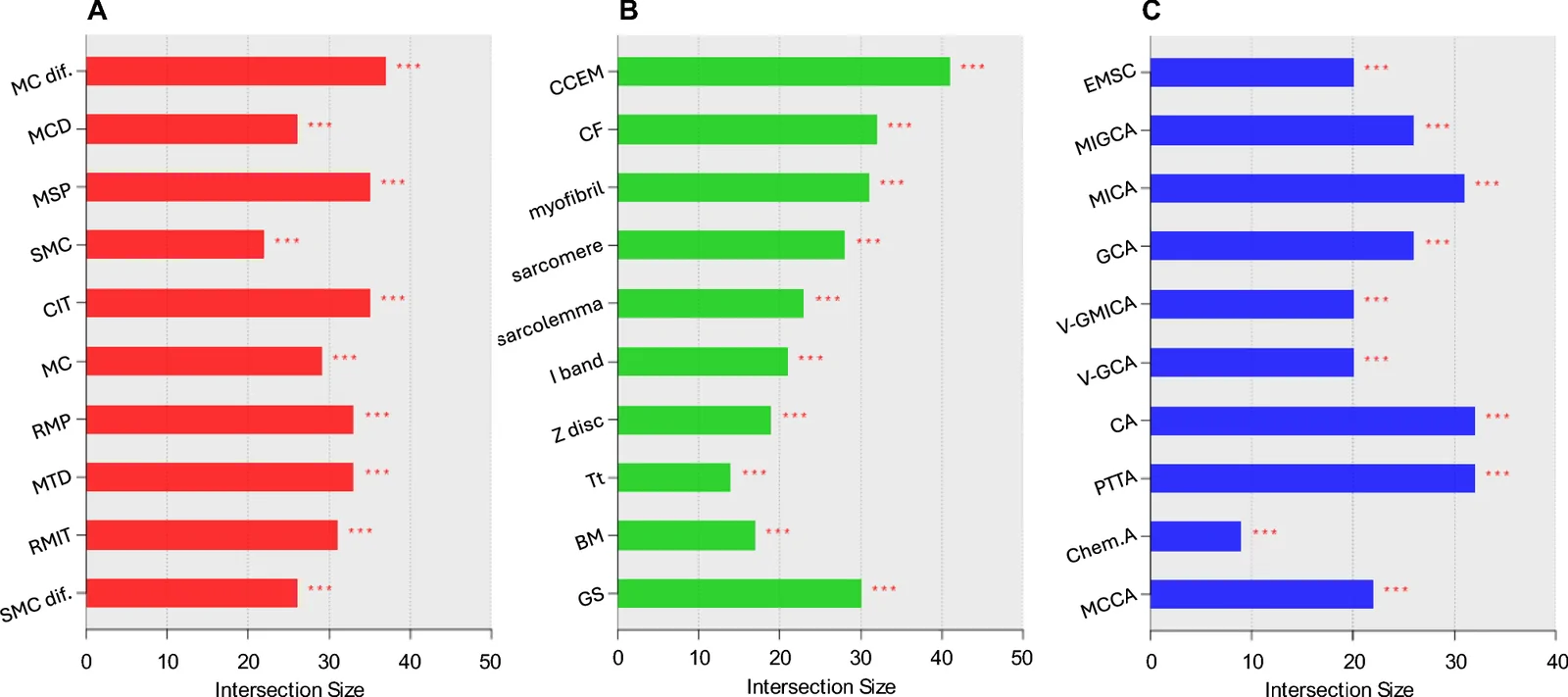
Top ten terms from Gene Ontology (GO) enrichment analysis in (**A**) biological process, (**B**) cellular component, and (**C**) molecular function domains. The top 10 significantly enriched GO terms are shown for each category. Terms with an adjusted ***p-value < 0.001 are marked as highly significant. MC dif., muscle cell differentiation; MCD, muscle cell development; MSP, muscle system process; SMC, striated muscle contraction; CIT, calcium ion transport; MC, muscle contraction; RMP, regulation of membrane potential; MTD, muscle tissue development; RMIT, regulation of metal ion transport; SMC dif., striated muscle cell differentiation; CCEM, collagen-containing extracellular matrix; CF, contractile fiber; BM, basement membrane; GS, glutamatergic synapse; EMSC, extracellular matrix structural constituent; MIGCA, monoatomic ion gated channel activity; MICA, monoatomic ion channel activity; GCA, gated channel activity; V-GMICA, voltage-gated monoatomic ion channel activity; V-GCA, voltage-gated channel activity; CA, channel activity; PTTA, passive transmembrane transporter activity; Chem.A, chemokine activity; MCCA, monoatomic cation channel activity.

To explore the functional significance of DEGs altered by combined exercise training, Gene Ontology (GO) enrichment analysis was conducted. The top 10 enriched GO terms from each domain (biological process, cellular component, and molecular function) were identified. In the biological process domain, DEGs were significantly enriched in terms related to “muscle cell differentiation, development, and contraction”, as well as “calcium and metal ion regulation” (Fig. 6A). In the cellular component domain, the top enriched term was "collagen-containing extracellular matrix," followed by “contractile fiber, myofibril”, and “glutamatergic synapse” (Fig. 6B). For the molecular function domain, enriched terms included "channel activity," "monoatomic ion channel activity," and "passive transmembrane transporter activity," suggesting exercise-induced modulation of ion transport across cellular membranes (Fig. 6C).

**Fig. 7:**
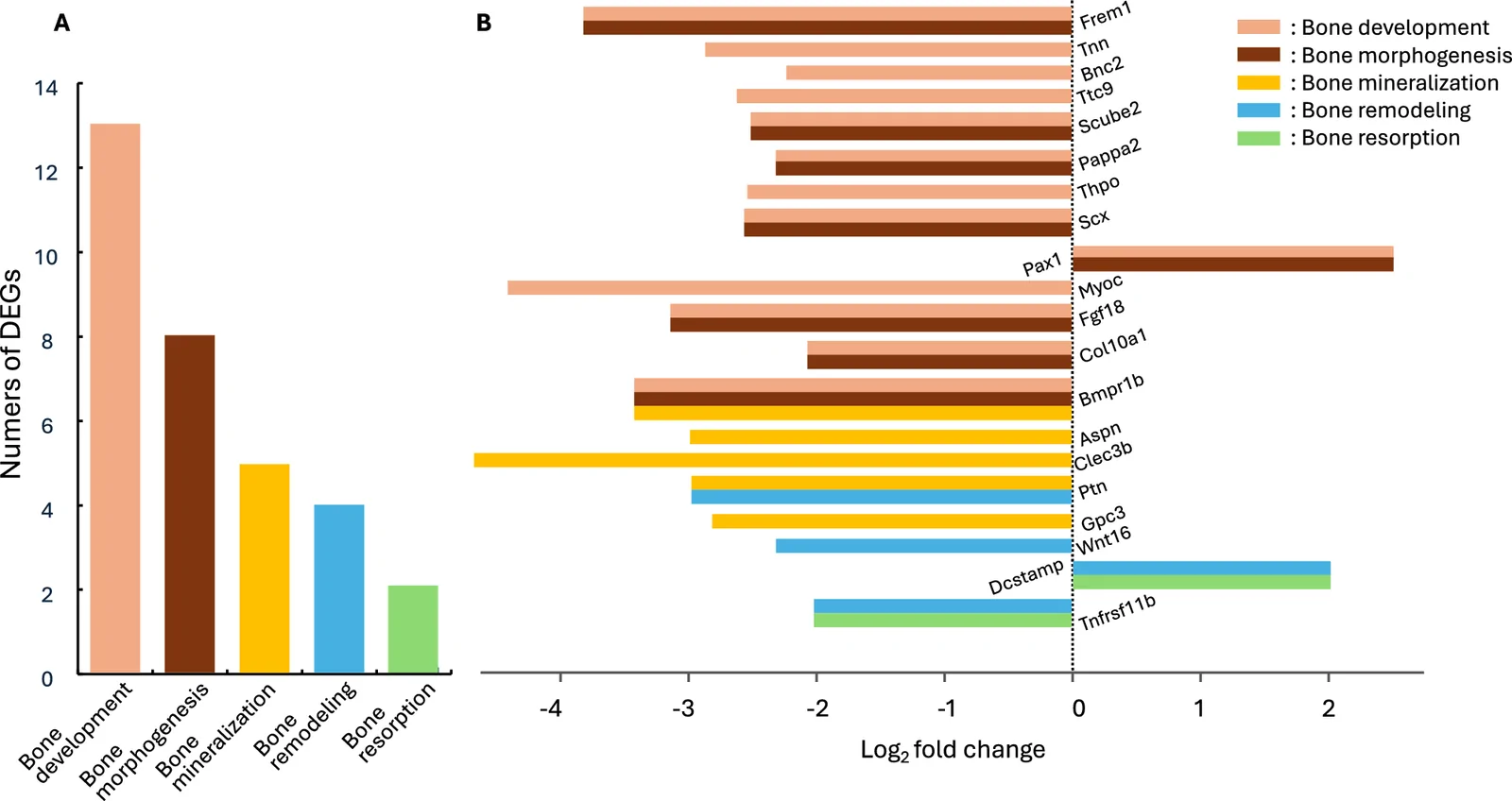
Bone metabolism-related Gene Ontology Biological Process (GO BP) terms enriched in DEGs following 12-week combined exercise training. (**A**) The number of DEGs associated with bone metabolism-related GO BP terms, including bone development (GO:0060348), bone morphogenesis (GO:0060349), bone mineralization (GO:0030282), bone remodeling (GO:0046849), and bone resorption (GO:0045453). (**B**) Differential expression (log₂ fold change) of 20 DEGs significantly altered by exercise across the five bone-related GO BP categories.

Given the focus of this study on bone tissue adaptations, we additionally examined GO Biological Process terms specifically related to bone metabolism. Among these, bone development (GO:0060348; adjusted *p* = 1.12×10⁻^2^) and bone morphogenesis (GO:0060349; adjusted *p* = 7.64×10⁻^3^) reached statistical significance, while bone mineralization, bone remodeling, and bone resorption showed enrichment without reaching adjusted significance (Fig. 7A; Supplementary Table 4). Within these bone-related GO categories, exercise training upregulated Pax1, a regulator of bone development and morphogenesis, and Dcstamp, involved in bone remodeling and resorption. Conversely, several bone development- and morphogenesis-related genes (*Fgf18*, *Scx*, *Scube2*, *Frem1*, *Col10a1*, *Pappa2*) and Tnfrsf11b, a key regulator of bone remodeling, were significantly downregulated (Fig. 7B).

**Fig. 8:**
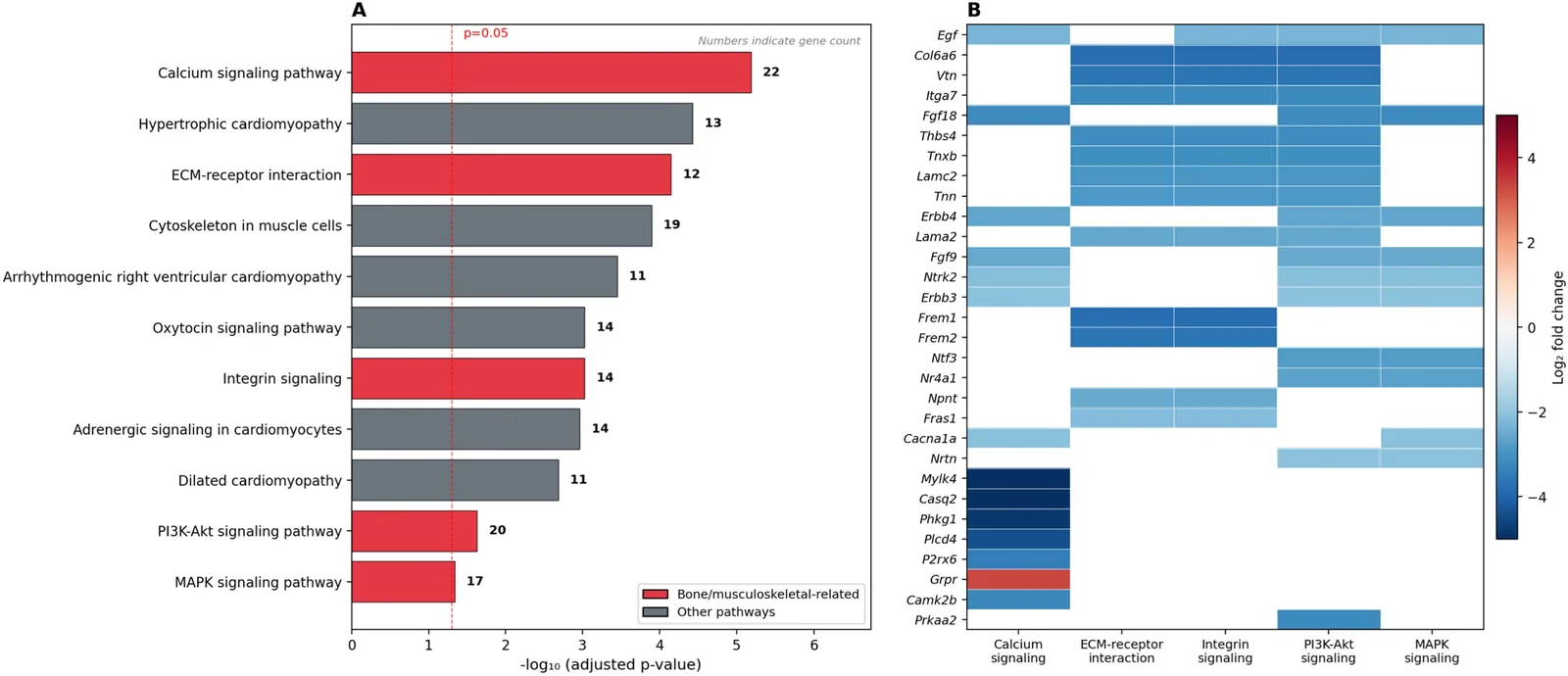
KEGG pathway enrichment analysis of differentially expressed genes (DEGs) following 12-week combined exercise training. (**A**) Eleven significantly enriched KEGG pathways identified through g:Profiler analysis (adjusted p < 0.05). Numbers next to each bar indicate the number of DEGs mapped to each pathway. Red bars indicate pathways with established roles in bone biology or musculoskeletal mechanotransduction. The red dashed line indicates the significance threshold (adjusted p = 0.05). (**B**) Heatmap showing log₂ fold change of DEGs mapped to the five bone-related KEGG pathways. Blue indicates downregulation; red indicates upregulation. Complete KEGG enrichment results are provided in Supplementary Table 5.

To further interrogate molecular signaling pathways underlying these transcriptomic changes, KEGG pathway enrichment analysis was performed. Eleven KEGG pathways were significantly enriched (adjusted p < 0.05; Fig. 8A), including several pathways with established roles in bone biology and mechanotransduction: Calcium signaling pathway (mmu04020; adjusted p = 6.54×10⁻⁶; 22 DEGs), ECM-receptor interaction (mmu04512; adjusted p = 7.10×10⁻^5^; 12 DEGs), Integrin signaling (mmu04518; adjusted p = 9.44×10⁻^4^; 14 DEGs), PI3K-Akt signaling pathway (mmu04151; adjusted p = 2.35×10⁻^2^; 20 DEGs), and MAPK signaling pathway (mmu04010; adjusted p = 4.58×10⁻^2^; 17 DEGs) (Fig. 8B). The complete list of significantly enriched KEGG pathways is provided in Supplementary Table 5.

## Discussion

Osteoporosis is characterized by reduced bone mineral density and deterioration of bone microarchitecture, increasing fracture susceptibility. Exercise is widely recognized as an effective non-pharmacological strategy for promoting bone health. However, the molecular mechanisms by which combined aerobic and resistance exercise modulate bone remodeling during peak bone mass acquisition remain poorly understood. In this study, 12 weeks of combined exercise training in early adult mice produced coordinated adaptations across multiple physiological domains. Combined exercise enhanced physical performance including grip strength and endurance capacity. It also induced favorable changes in body composition with attenuated body mass gain and increased heart and skeletal muscle mass relative to body weight. Exercise improved trabecular bone microarchitecture in the proximal tibia and altered the expression of 660 genes in bone tissue. To our knowledge, this is among the first studies to demonstrate that combined exercise not only improves trabecular bone structure but also modulates gene expression related to bone metabolism, providing novel molecular insight into exercise-induced skeletal adaptation during early adulthood.

The observed improvements in BV/TV, Tb.Th, and Tb.N, along with a trend toward reduced Tb.Sp, indicate a more interconnected and mechanically competent trabecular network. These findings are consistent with previous studies demonstrating that exercise training improves trabecular bone microarchitecture in rodent models^19, 28^. These structural adaptations are likely mediated through mechanotransduction pathways, as supported by the convergent findings from GO term enrichment and KEGG pathway analyses. GO enrichment highlighted calcium ion channel-related terms such as channel activity, monoatomic ion channel activity, and passive transmembrane transporter activity, and KEGG analysis further identified calcium signaling as the most significantly enriched pathway. The convergence of these complementary analyses strengthens the evidence that exercise-induced bone adaptation involves calcium-dependent signaling. Calcium ions serve as essential mediators of both muscle function and bone remodeling through their roles in osteoclast differentiation^29^ and matrix mineralization^30^. Dysregulated intracellular calcium levels have also been linked to increased bone resorption and osteoporosis pathogenesis^30^. Previous studies have demonstrated that intracellular calcium fluctuations regulate key osteoblastic signaling cascades, including the CaMKII^31^ and calcineurin–NFAT pathways^32^, both of which promote osteogenic gene expression and matrix synthesis. Furthermore, DEGs were significantly enriched in collagen-containing extracellular matrix, contractile fibers, and myofibrils within the cellular component category. This finding was paralleled by KEGG analysis identifying ECM-receptor interaction and integrin signaling as significantly enriched pathways. These convergent results suggest that exercise induces coordinated remodeling of the bone extracellular matrix and its receptor-mediated mechanosensing apparatus, consistent with the established role of integrins as mechanosensors in bone cells^33^. Together, these findings suggest that combined exercise initiates coordinated mechanotransduction-driven adaptation involving calcium-dependent signaling and ECM-integrin mechanosensing, although the precise mechanistic links warrant further investigation.

Beyond the skeletal adaptations, combined exercise also induced favorable changes in body composition and skeletal muscle mass. Despite the smaller body mass gain in the EXE group, normalized total skeletal muscle mass was significantly greater than in the CON group. This indicates that the attenuated body mass gain reflects reduced body fat accumulation rather than loss of lean tissue, consistent with the well-established role of exercise in promoting fat oxidation and lean tissue preservation. Among the individual skeletal muscles, soleus mass relative to body weight showed the most pronounced increase. The soleus is composed predominantly of oxidative slow-twitch fibers and is highly responsive to endurance-type training^34^, which likely explains its strong response to the aerobic component of the combined training program. In contrast, the plantaris and gastrocnemius, which contain a higher proportion of fast-twitch fibers^34^, showed only non-significant trends toward increased mass, suggesting that the resistance training stimulus may not have been sufficient to elicit significant hypertrophy of these muscles. Normalized heart mass was also significantly higher in the EXE group, consistent with exercise-induced physiological cardiac adaptation^35^. These coordinated adaptations in skeletal muscle, cardiac mass, and adiposity are consistent with the multisystem benefits of combined aerobic and resistance training and may further contribute to the observed improvements in bone microarchitecture through enhanced musculoskeletal mechanical coupling.

RNA sequencing-based transcriptomic analysis revealed significant transcriptional changes in response to combined exercise, consistent with previous transcriptomic studies demonstrating exercise-induced changes in bone gene expression^20^. These findings suggest that exercise may initiate transcriptional reprogramming associated with bone remodeling and metabolic adaptation, as evidenced by the concurrent upregulation of Dcstamp and Pax1, and downregulation of keratocan, Tnfrsf11b, and Fgf18. The most significantly downregulated gene was keratocan, an ECM protein expressed by osteoblast-lineage cells that plays a critical role in bone ECM composition and osteoblast-mediated mineralization^36^. Keratocan-deficient mice exhibit impaired mineralization and reduced expression of osteogenic markers such as osteocalcin and bone sialoprotein^36^. Fragmentation of keratocan and other SLRPs has been observed in degenerated articular cartilage and meniscal tissues^37^. Thus, the observed downregulation may reflect a remodeling-driven shift in ECM composition during exercise-induced adaptation, rather than a simple suppression of osteogenic potential, as the role of keratocan in ECM maintenance appears to be dynamic and context-dependent. Among the most highly upregulated genes were members of the immunoglobulin kappa variable (Igkv) family and formyl peptide receptor 3 (Fpr3), suggesting exercise-induced modulation of the bone marrow immune microenvironment. Igkv genes encode components of immunoglobulin light chains involved in immune responses within the bone marrow niche, and Fpr3 is a pattern recognition receptor expressed on immune cells^38^, which may participate in resolving exercise-induced inflammation within bone tissue. Furthermore, exercise-induced regulation of immune-related genes in bone may reflect modulation of the peri-arteriolar niche, where mechanical stimulation has been shown to maintain lymphoid progenitors alongside osteogenic cells^39^. These observations suggest coordinated changes in the bone marrow microenvironment through modulation of osteoclast activity, cytokine production, and stromal-immune cell communication^14^.

GO Biological Process enrichment analysis specifically focused on bone metabolism identified several key genes that were significantly modulated by combined exercise including Pax1, Dcstamp, and Tnfrsf11b. Pax1 expression was increased in the EXE group; although Pax1 has been primarily studied in the context of embryonic vertebral column development^40, 41^, its upregulation in adult bone tissue in the present study suggests a potential role in exercise-induced postnatal bone remodeling. Similarly, Dcstamp, which encodes a transmembrane protein essential for osteoclast precursor fusion and bone resorption^42^, was upregulated in the EXE group. The regulation of these bone-related genes is likely mediated through the molecular signaling cascades identified in our KEGG analysis, in which PI3K-Akt and MAPK signaling pathways, both well-established regulators of osteoblast differentiation, survival, and osteoclast activity^43,^^44^ were significantly enriched, providing mechanistic context for the observed changes in bone-related gene expression. Dcstamp-deficient mice fail to form multinucleated osteoclasts, resulting in osteopetrosis^42, 45^, underscoring its critical role in osteoclastogenesis and skeletal homeostasis. DC-STAMP has been established as an essential regulator of osteoclast precursor fusion and bone remodeling^46^, and its upregulation has been associated with enhanced bone resorption activity in vivo^47^. In contrast, Tnfrsf11b, encoding osteoprotegerin (OPG), was downregulated following exercise. OPG functions as a decoy receptor for RANKL, suppressing osteoclastogenesis and bone resorption^48^. While exercise has been reported to modulate the RANKL/RANK/OPG axis in ways dependent on exercise type, intensity, and duration^49^, the observed reduction in Tnfrsf11b expression likely reflects a transient decrease in anti-resorptive signaling. When interpreted alongside the upregulation of osteoclast-related genes and improvements in bone microarchitecture, this change suggests an adaptive and coordinated remodeling response rather than pathological bone loss.

Our findings demonstrate that combined aerobic and resistance exercise induces multifaceted molecular adaptations in bone tissue, encompassing ECM remodeling, osteoimmune signaling, calcium homeostasis, and gene networks associated with bone remodeling. The convergent findings from GO and KEGG analyses, particularly the enrichment of calcium signaling, ECM-receptor interaction, integrin signaling, and PI3K-Akt and MAPK pathways, collectively support a mechanotransduction-driven model of exercise-induced bone adaptation. In particular, the concurrent modulation of Pax1, Dcstamp, and Tnfrsf11b highlights a coordinated transcriptional response involving both osteogenic and osteoclastic pathways, suggesting a potentially novel regulatory mechanism in which exercise simultaneously activates osteoclast precursor fusion (via Dcstamp upregulation), modulates skeletal patterning factors (via Pax1 upregulation), and suppresses anti-resorptive signaling (via Tnfrsf11b downregulation), collectively reflecting a coordinated transcriptional response to mechanical loading. These molecular adaptations may underlie the observed improvements in trabecular bone microarchitecture during early adulthood and reinforce the role of exercise as an effective non-pharmacological strategy to support long-term bone health and mitigate future osteoporosis risk. Nonetheless, several limitations should be acknowledged. The sample size for the overall study (n=8 per group) was consistent with prior rodent exercise studies, but the RNA-seq analysis used a subset of n=4 mice per group, which may have limited the statistical power for detecting smaller transcriptomic changes. Further validation using qRT-PCR and protein-level analyses, along with expanded studies involving larger and more diverse populations and extended training durations, is warranted. In addition, μCT analysis was performed at a single endpoint following tissue harvest, rather than longitudinally across the intervention period, due to practical and ethical considerations regarding repeated anesthesia exposure. Future studies incorporating time-course in vivo μCT measurements would provide valuable insight into the temporal progression of exercise-induced trabecular and cortical bone adaptations. Calcein double-labeling was also not performed in this study, which would have enabled direct assessment of dynamic bone formation parameters such as mineral apposition rate and bone formation rate. Future studies incorporating calcein labeling and cross-sectional cortical bone imaging would provide more comprehensive insight into exercise-induced bone formation dynamics.

## Supplementary Information


Supplementary Information 1.
Supplementary Information 2.
Supplementary Information 3.
Supplementary Information 4.
Supplementary Information 5.


## Data Availability

All data generated or analyzed during this study are included in this published article and its supplementary information files.
