# Supplementary material for: Transcriptomic insights into exercise-induced trabecular bone microarchitectural adaptations following combined aerobic and resistance training in mice
Source: Sci Rep. 2026 Jun 15;16:27219. doi: 10.1038/s41598-026-56753-6 (PMC13527104; doi:10.1038/s41598-026-56753-6)
Supplement: Supplementary file 1 [file 41598_2026_56753_MOESM1_ESM.docx]

**Supplementary Table 1.**

**Supplementary Table 1.** Aerobic treadmill exercise protocol.

| **Weeks** | **Warm-up** | **Main Exercise** | | **Cool-down** | **Total time** |
| --- | --- | --- | --- | --- | --- |
|  |  | **Speed** | **Time** |  |  |
| 0 | 0 m/min | 5 m/min | 5 min | 5 min | 10 min |
| 1 | 5 m/min | 8 m/min | 20 min | 5 min | 30 min |
| 2 | 5 m/min | 10 m/min | 20 min | 5 min | 30 min |
| 3 | 5 m/min | 12 m/min | 25 min | 5 min | 35 min |
| 4 | 5 m/min | 14 m/min | 25 min | 5 min | 35 min |
| 5 | 5 m/min | 16 m/min | 30 min | 5 min | 40 min |
| 6 | 5 m/min | 18 m/min | 30 min | 5 min | 40 min |
| 7 | 5 m/min | 18 m/min | 35 min | 5 min | 45 min |
| 8 | 5 m/min | 18 m/min | 35 min | 5 min | 45 min |
| 9 | 5 m/min | 18 m/min | 40 min | 5 min | 50 min |
| 10 | 5 m/min | 18 m/min | 40 min | 5 min | 50 min |
| 11 | 5 m/min | 18 m/min | 40 min | 5 min | 50 min |
| 12 | 5 m/min | 18 m/min | 40 min | 5 min | 50 min |
