# Supplementary material for: Transcriptomic insights into exercise-induced trabecular bone microarchitectural adaptations following combined aerobic and resistance training in mice
Source: Sci Rep. 2026 Jun 15;16:27219. doi: 10.1038/s41598-026-56753-6 (PMC13527104; doi:10.1038/s41598-026-56753-6)
Supplement: Supplementary file 2 [file 41598_2026_56753_MOESM2_ESM.docx]

**Supplementary Table 2.**

| **Weeks** | **Load (%BW)** | **Repetition** | **Set** | | **Total reps** | **Rest time** |
| --- | --- | --- | --- | --- | --- | --- |
| 0 | 0 | 1-3 | | 1 | 1-3 | 0 |
| 1 | 50% | 3 | | 2 | 6 | 2 min |
| 2 | 60% | 3 | | 2 | 6 | 2 min |
| 3 | 70% | 3 | | 2 | 6 | 2 min |
| 4 | 80% | 3 | | 2 | 6 | 2 min |
| 5 | 90% | 3 | | 3 | 9 | 2 min |
| 6 | 100% | 3 | | 3 | 9 | 2 min |
| 7 | 110% | 3 | | 3 | 9 | 2 min |
| 8 | 120% | 3 | | 3 | 12 | 2 min |
| 9 | 130% | 3 | | 4 | 12 | 2 min |
| 10 | 140% | 3 | | 4 | 12 | 2 min |
| 11 | 150% | 3 | | 4 | 12 | 2 min |
| 12 | 160% | 3 | | 4 | 12 | 2 min |

**Supplementary Table 2.** Ladder climbing resistance exercise protocol.
